# Di-μ_2_-methoxo-bis­{[μ-3,10,18,25-tetra­aza­penta­cyclo­[17.4.4.3.1.1]triconta-1(31),2,4(9),5,7,10,12,14,16(32),17,19(24),20,22,25,27,29-hexa­deca­ene-31,32-diolato]dizinc(II)} bis­(perchlorate) *N*,*N*-dimethyl­formamide disolvate

**DOI:** 10.1107/S1600536811005873

**Published:** 2011-02-23

**Authors:** Zelong Lim, Craig M. Forsyth, Bim Graham

**Affiliations:** a Medicinal Chemistry and Drug Action, Monash Institute of Pharmaceutical Sciences, Monash University (Parkville Campus), 381 Royal Parade, Parkville, Victoria 3052, Australia; b School of Chemistry, Monash University, Clayton, Victoria 3800, Australia

## Abstract

The title compound, [Zn_4_(C_28_H_18_N_4_O_2_)_2_(CH_3_O)_2_](ClO_4_)_2_·2C_3_H_7_NO, is a *C*2 symmetric tetra­nuclear zinc(II) complex comprised of two [Zn_2_ *L*]^2+^ units bridged by a pair of μ_2_-OMe ligands (where *L* is the doubly-deprotonated form of the macrocyclic dinucleating ligand derived from the [2 + 2] Schiff base condensation between 2-hy­droxy­benzene-1,3-dicarbaldehyde and 1,2-diamino­benzene). Each Zn^II^ atom has a distorted square-pyramidal coordination geometry and the Zn_4_(μ-OMe)_2_ unit lies in the cleft formed by two distinctly bent Schiff base ligands. The observed mol­ecular shape is supported by an intra­molecular π–π inter­action between one of the phenolate rings on each of the two ligands [centroid–centroid distance = 3.491 (5) Å]. The methyl groups of the solvent molecule are disordered over two sets of sites in a 0.6:0.4 ratio.

## Related literature

For the first examples of polynuclear transition metal complexes of Schiff base macrocyclic ligands, see: Pilkington & Robson (1970 ▶). For complexes comprising of macrocyclic ligands derived from 2-hy­droxy-benzene-1,3-dicarbaldehyde and diamines or triamines, see: Vigato *et al.* (1990 ▶, 2007 ▶); Huang *et al.* (2006 ▶).
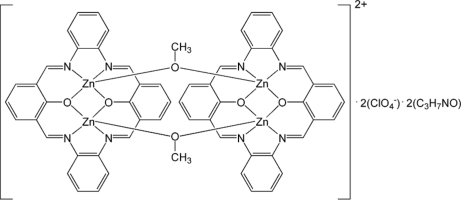


## Experimental

### 

#### Crystal data

[Zn_4_(C_28_H_18_N_4_O_2_)_2_(CH_3_O)_2_](ClO_4_)_2_·2C_3_H_7_NO
*M* *_r_* = 1553.57
Monoclinic, 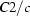

*a* = 30.9454 (4) Å
*b* = 10.4512 (2) Å
*c* = 20.5774 (4) Å
β = 112.019 (1)°
*V* = 6169.65 (19) Å^3^
*Z* = 4
Mo *K*α radiation
μ = 1.70 mm^−1^
*T* = 123 K
0.20 × 0.20 × 0.13 mm

#### Data collection

Nonius KappaCCD diffractometer
Absorption correction: multi-scan (*SORTAV*; Blessing, 1997 ▶) *T*_min_ = 0.92, *T*_max_ = 1.0
32276 measured reflections
6063 independent reflections
4245 reflections with *I* > 2σ(*I*)
*R*_int_ = 0.074

#### Refinement

*R*[*F* ^2^ > 2σ(*F* ^2^)] = 0.045
*wR*(*F* ^2^) = 0.123
*S* = 1.03
6063 reflections
453 parameters
17 restraints
H-atom parameters constrained
Δρ_max_ = 0.86 e Å^−3^
Δρ_min_ = −0.47 e Å^−3^

### 

Data collection: *COLLECT* (Nonius, 2004 ▶); cell refinement: *DENZO-SMN* (Otwinowski & Minor, 1997) ▶; data reduction: *DENZO-SMN* ▶; program(s) used to solve structure: *SHELXS97* (Sheldrick, 2008 ▶); program(s) used to refine structure: *SHELXL97* (Sheldrick, 2008 ▶); molecular graphics: *X-SEED* (Barbour, 2001 ▶); software used to prepare material for publication: *CIFTAB* (Sheldrick, 1997 ▶).

## Supplementary Material

Crystal structure: contains datablocks global, I. DOI: 10.1107/S1600536811005873/im2265sup1.cif

Structure factors: contains datablocks I. DOI: 10.1107/S1600536811005873/im2265Isup2.hkl

Additional supplementary materials:  crystallographic information; 3D view; checkCIF report

## Figures and Tables

**Fig. 1.**
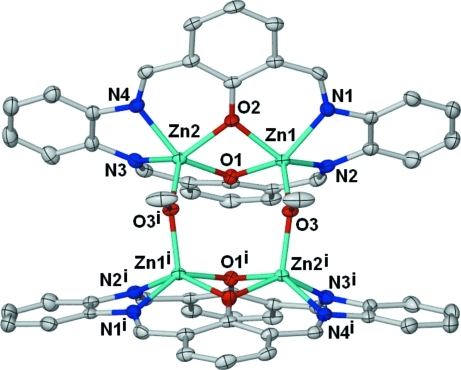
A view of the complex cation of (I) showing labeling of selected non-hydrogen atoms. Displacement ellipsoids are drawn at the 50% probability level and hydrogen atoms have been omitted for clarity. Atoms denoted 'i' are generated by the symmetry operator: 1 - x, y, 0.5 - z.

## supplementary crystallographic information

### Comment

The molecular structure of [Zn_4_*L*_2_(CH_3_O)_2_](ClO_4_)_2_ × 2 DMF (1) (Figure 1) features two Schiff base ligands, each of which coordinates a pair of zinc(II) centres. The dinuclear subunits are then bridged by two exogenous methoxo ligands. The two independent zinc(II) atoms have similar square-pyramidal coordination environments. The basal plane consists of nitrogen and oxygen atoms of the Schiff base ligand (mean deviation Zn1 0.004 (3) Å, Zn2 0.019 (3) Å); Zn1 and Zn2 lie out of these basal planes by 0.644 (1) and 0.654 (1) Å, respectively. The oxygen atoms, O3 and O3^i^ (symmetry code: i 1 - *x*, *y*, 0.5 - *z*), of the bridging methoxo ligands occupy the apical positions. The two zinc(II) centres within each macrocyclic cavity are separated by 3.0136 (6) Å, whilst those bridged by the methoxo ligand are situated 3.4458 (5) Å apart.

Within the cation, there is an aromatic π-π interaction between the phenolic ring (C8—C13) from each of the Schiff base ligands. The centroid-centroid separation between them is 3.491 (5) Å and there is a distinct bending of the Schiff base ligand with the C8—C13 ring forming an angle of 27.44 (9) ° to the N1, N2, N3, N4 plane (*cf* 4.1 (1) ° for the other phenolic ring C15—C20). Overall, the cation adopts a 'cleft-shaped' structure with the associated ClO_4_^-^ anions and DMF molecules located near the periphery of the cleft opening.

### Experimental

A solution of 2-hydroxy-benzene-1,3-dicarbaldehyde (2 mmol) in ethanol (10 ml) was added dropwise to a stirred solution of Zn(ClO_4_)_2_ × 6 H_2_O (2 mmol) and 1,2-diaminobenzene (2 mmol) in ethanol (20 ml). Triethylamine (2 mmol) was then added and the reaction mixture was stirred for 3 h at room temperature. The solvent was removed *in vacuo* and the resulting solid was recrystallized by slow diffusion of methanol into an *N,N*-dimethylformamide solution to give the title compound as yellow blocks in 64% yield. IR (cm^-1^): 622 (ClO_4_), 1090 (ClO_4_), 1540 (C=C), 1615 (C=N). CAUTION: Although no problems were encountered in this work transition metal perchlorates are potentially explosive. They should be prepared in small quantities and handled with care.

### Refinement

All H atoms were placed in geometrically idealized positions and constrained to ride on their parent atoms with C—H distances in the range 0.95–1.00 Å and *U*_iso_(H) = 1.2–1.5 *U*_eq_(C).

The methyl groups of the solvent DMF molecule, C31 and C32, were modeled as disordered over two positions 0.8 Å apart (refinined occupancies 0.60:0.40) and were refined with constrained N—C distances and anisotropic thermal parameters.

### Crystal data

**Table:**

[Zn_4_(C_28_H_18_N_4_O_2_)_2_(CH_3_O)_2_](ClO_4_)_2_·2C_3_H_7_NO	*F*(000) = 3168
*M**_r_* = 1553.57	*D*_x_ = 1.673 Mg m^−^^3^
Monoclinic, *C*2/*c*	Mo *K*α radiation, λ = 0.71073 Å
Hall symbol: -C 2yc	Cell parameters from 3613 reflections
*a* = 30.9454 (4) Å	θ = 2.1–26.0°
*b* = 10.4512 (2) Å	µ = 1.70 mm^−^^1^
*c* = 20.5774 (4) Å	*T* = 123 K
β = 112.019 (1)°	Block, yellow
*V* = 6169.65 (19) Å^3^	0.20 × 0.20 × 0.13 mm
*Z* = 4	

### Data collection

**Table:**

Nonius KappaCCD diffractometer	6063 independent reflections
Radiation source: fine-focus sealed tube	4245 reflections with *I* > 2σ(*I*)
horizonally mounted graphite crystal	*R*_int_ = 0.074
Detector resolution: 9 pixels mm^-1^	θ_max_ = 26.0°, θ_min_ = 2.1°
Thin–slice φ and ω scans	*h* = −38→38
Absorption correction: multi-scan (*SORTAV*; Blessing, 1997)	*k* = −12→12
*T*_min_ = 0.92, *T*_max_ = 1.0	*l* = −25→25
32276 measured reflections	

### Refinement

**Table:**

Refinement on *F*^2^	Primary atom site location: structure-invariant direct methods
Least-squares matrix: full	Secondary atom site location: difference Fourier map
*R*[*F*^2^ > 2σ(*F*^2^)] = 0.045	Hydrogen site location: inferred from neighbouring sites
*wR*(*F*^2^) = 0.123	H-atom parameters constrained
*S* = 1.03	*w* = 1/[σ^2^(*F*_o_^2^) + (0.0611*P*)^2^ + 14.7497*P*] where *P* = (*F*_o_^2^ + 2*F*_c_^2^)/3
6063 reflections	(Δ/σ)_max_ = 0.001
453 parameters	Δρ_max_ = 0.86 e Å^−^^3^
17 restraints	Δρ_min_ = −0.47 e Å^−^^3^

### Special details

**Table:**

Geometry. All e.s.d.'s (except the e.s.d. in the dihedral angle between two l.s. planes) are estimated using the full covariance matrix. The cell e.s.d.'s are taken into account individually in the estimation of e.s.d.'s in distances, angles and torsion angles; correlations between e.s.d.'s in cell parameters are only used when they are defined by crystal symmetry. An approximate (isotropic) treatment of cell e.s.d.'s is used for estimating e.s.d.'s involving l.s. planes.
Refinement. Refinement of *F*^2^ against ALL reflections. The weighted *R*-factor *wR* and goodness of fit *S* are based on *F*^2^, conventional *R*-factors *R* are based on *F*, with *F* set to zero for negative *F*^2^. The threshold expression of *F*^2^ > σ(*F*^2^) is used only for calculating *R*-factors(gt) *etc*. and is not relevant to the choice of reflections for refinement. *R*-factors based on *F*^2^ are statistically about twice as large as those based on *F*, and *R*- factors based on ALL data will be even larger.The methyl groups of the solvent DMF molecule, C31 and C32, were modeled as disordered over two positions 0.8 Å apart (refined occupancies 0.60:0.40) and were refined with constrained N—C distances and anisotropic thermal parameters.

### Fractional atomic coordinates and isotropic or equivalent isotropic displacement parameters (Å^2^)

**Table:**

	*x*	*y*	*z*	*U*_iso_*/*U*_eq_	Occ. (<1)
Zn1	0.544502 (14)	0.56311 (4)	0.36676 (2)	0.01940 (13)	
Zn2	0.440196 (15)	0.56125 (4)	0.29071 (2)	0.01988 (13)	
Cl3	0.84640 (4)	0.42735 (11)	0.41666 (5)	0.0349 (3)	
O1	0.49269 (8)	0.6907 (2)	0.31948 (12)	0.0221 (6)	
O2	0.48877 (9)	0.4672 (2)	0.37215 (13)	0.0246 (6)	
O3	0.56028 (9)	0.4799 (2)	0.29429 (12)	0.0211 (6)	
O4	0.66347 (13)	−0.0167 (4)	0.36447 (19)	0.0604 (10)	
O5	0.80381 (13)	0.4340 (5)	0.4259 (2)	0.0914 (17)	
O6	0.84744 (12)	0.5253 (4)	0.3665 (2)	0.0599 (10)	
O7	0.88417 (12)	0.4450 (4)	0.48234 (17)	0.0586 (10)	
O8	0.85150 (17)	0.3080 (4)	0.38887 (19)	0.0873 (15)	
N1	0.58342 (11)	0.4818 (3)	0.46159 (16)	0.0232 (7)	
N2	0.58800 (11)	0.7109 (3)	0.40917 (16)	0.0240 (7)	
N3	0.39402 (11)	0.7064 (3)	0.27061 (16)	0.0239 (7)	
N4	0.39069 (11)	0.4756 (3)	0.32117 (16)	0.0236 (7)	
N5	0.68833 (17)	0.1882 (4)	0.3794 (3)	0.0628 (13)	
C1	0.62745 (13)	0.5440 (4)	0.48975 (19)	0.0245 (9)	
C2	0.63005 (13)	0.6653 (4)	0.46105 (19)	0.0256 (9)	
C3	0.67245 (15)	0.7315 (5)	0.4850 (2)	0.0376 (11)	
H3	0.6747	0.8128	0.4658	0.045*	
C4	0.71091 (15)	0.6788 (5)	0.5362 (2)	0.0424 (12)	
H4	0.7395	0.7245	0.5524	0.051*	
C5	0.70858 (15)	0.5603 (5)	0.5645 (2)	0.0374 (11)	
H5	0.7355	0.5256	0.5999	0.045*	
C6	0.66721 (14)	0.4916 (4)	0.5414 (2)	0.0319 (10)	
H6	0.6658	0.4096	0.5605	0.038*	
C7	0.57915 (14)	0.8310 (4)	0.3979 (2)	0.0266 (9)	
H7	0.6043	0.8888	0.4185	0.032*	
C8	0.53386 (14)	0.8859 (4)	0.35607 (19)	0.0230 (8)	
C9	0.49187 (13)	0.8162 (4)	0.32477 (18)	0.0214 (8)	
C10	0.44868 (14)	0.8847 (4)	0.30100 (18)	0.0243 (9)	
C11	0.44939 (15)	1.0188 (4)	0.3043 (2)	0.0306 (10)	
H11	0.4207	1.0642	0.2884	0.037*	
C12	0.49064 (15)	1.0866 (4)	0.3302 (2)	0.0322 (10)	
H12	0.4904	1.1774	0.3295	0.039*	
C13	0.53222 (16)	1.0211 (4)	0.3570 (2)	0.0304 (10)	
H13	0.5605	1.0679	0.3765	0.037*	
C14	0.40247 (14)	0.8270 (4)	0.27734 (19)	0.0270 (9)	
H14	0.3765	0.8832	0.2660	0.032*	
C15	0.34817 (13)	0.6556 (4)	0.2554 (2)	0.0270 (9)	
C16	0.34680 (13)	0.5339 (4)	0.2840 (2)	0.0260 (9)	
C17	0.30367 (14)	0.4767 (5)	0.2720 (2)	0.0348 (10)	
H17	0.3022	0.3953	0.2916	0.042*	
C18	0.26309 (16)	0.5393 (5)	0.2314 (2)	0.0390 (11)	
H18	0.2337	0.5008	0.2235	0.047*	
C19	0.26487 (15)	0.6566 (5)	0.2025 (2)	0.0409 (12)	
H19	0.2367	0.6974	0.1739	0.049*	
C20	0.30707 (15)	0.7164 (4)	0.2143 (2)	0.0356 (10)	
H20	0.3079	0.7981	0.1945	0.043*	
C21	0.39648 (14)	0.3803 (4)	0.36304 (19)	0.0256 (9)	
H21	0.3692	0.3429	0.3658	0.031*	
C22	0.44099 (13)	0.3244 (4)	0.40659 (19)	0.0239 (9)	
C23	0.48531 (13)	0.3777 (4)	0.41457 (19)	0.0228 (8)	
C24	0.52560 (14)	0.3311 (4)	0.46991 (19)	0.0256 (9)	
C25	0.52120 (15)	0.2229 (4)	0.5082 (2)	0.0285 (9)	
H25	0.5482	0.1902	0.5444	0.034*	
C26	0.47916 (15)	0.1639 (4)	0.4945 (2)	0.0310 (10)	
H26	0.4775	0.0872	0.5182	0.037*	
C27	0.43931 (15)	0.2168 (4)	0.4462 (2)	0.0281 (9)	
H27	0.4100	0.1794	0.4395	0.034*	
C28	0.57110 (14)	0.3906 (4)	0.49350 (19)	0.0262 (9)	
H28	0.5939	0.3601	0.5361	0.031*	
C29	0.5691 (2)	0.3485 (4)	0.3017 (2)	0.0519 (14)	
H29A	0.5406	0.3016	0.2747	0.078*	
H29B	0.5793	0.3250	0.3513	0.078*	
H29C	0.5936	0.3269	0.2842	0.078*	
C30	0.67208 (19)	0.0813 (6)	0.3976 (3)	0.0556 (15)	
H30	0.6669	0.0828	0.4403	0.067*	
C31	0.7049 (6)	0.2897 (15)	0.4349 (9)	0.084 (4)	0.60 (2)
H31A	0.7168	0.3629	0.4169	0.126*	0.60 (2)
H31B	0.7299	0.2550	0.4763	0.126*	0.60 (2)
H31C	0.6789	0.3174	0.4478	0.126*	0.60 (2)
C32	0.6894 (5)	0.1867 (13)	0.3083 (6)	0.056 (3)	0.60 (2)
H32A	0.7017	0.2683	0.2992	0.084*	0.60 (2)
H32B	0.6577	0.1740	0.2737	0.084*	0.60 (2)
H32C	0.7094	0.1167	0.3047	0.084*	0.60 (2)
C31'	0.6860 (5)	0.3255 (11)	0.4007 (12)	0.055 (5)	0.40 (2)
H31D	0.7025	0.3803	0.3789	0.082*	0.40 (2)
H31E	0.7007	0.3329	0.4518	0.082*	0.40 (2)
H31F	0.6533	0.3523	0.3852	0.082*	0.40 (2)
C32'	0.7093 (8)	0.1998 (19)	0.3261 (9)	0.053 (5)	0.40 (2)
H32D	0.7171	0.2896	0.3220	0.080*	0.40 (2)
H32E	0.6871	0.1696	0.2808	0.080*	0.40 (2)
H32F	0.7377	0.1479	0.3400	0.080*	0.40 (2)

### Atomic displacement parameters (Å^2^)

**Table:**

	*U*^11^	*U*^22^	*U*^33^	*U*^12^	*U*^13^	*U*^23^
Zn1	0.0232 (2)	0.0180 (2)	0.0188 (2)	−0.00098 (19)	0.00989 (18)	−0.00027 (17)
Zn2	0.0236 (2)	0.0188 (2)	0.0191 (2)	0.00050 (19)	0.01015 (18)	−0.00067 (17)
Cl3	0.0274 (5)	0.0398 (6)	0.0327 (5)	−0.0016 (5)	0.0057 (4)	−0.0034 (5)
O1	0.0236 (14)	0.0164 (14)	0.0265 (14)	−0.0006 (11)	0.0095 (11)	−0.0011 (11)
O2	0.0236 (14)	0.0262 (15)	0.0252 (14)	−0.0023 (12)	0.0104 (12)	0.0083 (11)
O3	0.0287 (15)	0.0167 (13)	0.0220 (13)	0.0045 (11)	0.0143 (12)	0.0041 (10)
O4	0.070 (3)	0.049 (2)	0.060 (2)	−0.018 (2)	0.022 (2)	−0.0024 (19)
O5	0.034 (2)	0.174 (5)	0.064 (3)	0.005 (3)	0.0168 (19)	0.042 (3)
O6	0.044 (2)	0.063 (3)	0.065 (2)	−0.0124 (18)	0.0112 (18)	0.0204 (19)
O7	0.041 (2)	0.085 (3)	0.0386 (19)	0.0051 (19)	0.0016 (16)	−0.0150 (18)
O8	0.148 (4)	0.048 (2)	0.045 (2)	0.012 (3)	0.012 (2)	−0.0124 (19)
N1	0.0244 (18)	0.0235 (18)	0.0214 (16)	0.0011 (14)	0.0083 (14)	−0.0013 (13)
N2	0.0283 (18)	0.0236 (19)	0.0238 (16)	−0.0044 (15)	0.0141 (14)	−0.0020 (13)
N3	0.0260 (18)	0.0233 (19)	0.0216 (16)	0.0037 (15)	0.0079 (14)	0.0001 (13)
N4	0.0251 (18)	0.0249 (18)	0.0237 (17)	−0.0037 (14)	0.0123 (14)	−0.0042 (14)
N5	0.080 (3)	0.049 (3)	0.087 (3)	−0.022 (3)	0.063 (3)	−0.023 (2)
C1	0.024 (2)	0.033 (2)	0.0184 (18)	−0.0023 (17)	0.0112 (16)	−0.0079 (16)
C2	0.026 (2)	0.032 (2)	0.0217 (19)	−0.0006 (18)	0.0121 (17)	−0.0037 (17)
C3	0.036 (3)	0.043 (3)	0.033 (2)	−0.012 (2)	0.012 (2)	−0.003 (2)
C4	0.028 (2)	0.064 (3)	0.034 (2)	−0.015 (2)	0.011 (2)	−0.006 (2)
C5	0.027 (2)	0.057 (3)	0.028 (2)	0.001 (2)	0.0106 (19)	−0.001 (2)
C6	0.033 (2)	0.039 (3)	0.023 (2)	0.007 (2)	0.0098 (18)	−0.0001 (19)
C7	0.031 (2)	0.026 (2)	0.028 (2)	−0.0110 (18)	0.0171 (18)	−0.0076 (17)
C8	0.036 (2)	0.019 (2)	0.0206 (19)	−0.0018 (18)	0.0183 (17)	0.0006 (15)
C9	0.033 (2)	0.0176 (19)	0.0182 (18)	0.0005 (17)	0.0147 (16)	0.0007 (15)
C10	0.039 (2)	0.017 (2)	0.0196 (19)	0.0014 (18)	0.0147 (18)	0.0024 (15)
C11	0.044 (3)	0.023 (2)	0.029 (2)	0.010 (2)	0.018 (2)	0.0056 (17)
C12	0.052 (3)	0.019 (2)	0.034 (2)	0.001 (2)	0.026 (2)	0.0038 (18)
C13	0.047 (3)	0.024 (2)	0.029 (2)	−0.006 (2)	0.023 (2)	−0.0013 (17)
C14	0.034 (2)	0.026 (2)	0.023 (2)	0.0091 (19)	0.0133 (17)	0.0036 (17)
C15	0.024 (2)	0.031 (2)	0.025 (2)	0.0039 (18)	0.0075 (17)	−0.0062 (17)
C16	0.025 (2)	0.033 (2)	0.022 (2)	0.0021 (18)	0.0106 (17)	−0.0069 (16)
C17	0.031 (2)	0.043 (3)	0.031 (2)	−0.003 (2)	0.0134 (19)	−0.0067 (19)
C18	0.028 (2)	0.054 (3)	0.034 (2)	−0.002 (2)	0.010 (2)	−0.009 (2)
C19	0.028 (2)	0.054 (3)	0.035 (2)	0.012 (2)	0.006 (2)	−0.005 (2)
C20	0.037 (3)	0.038 (3)	0.028 (2)	0.009 (2)	0.0090 (19)	−0.0039 (19)
C21	0.029 (2)	0.027 (2)	0.026 (2)	−0.0064 (18)	0.0163 (18)	−0.0072 (17)
C22	0.031 (2)	0.020 (2)	0.026 (2)	−0.0016 (17)	0.0168 (17)	−0.0017 (16)
C23	0.034 (2)	0.017 (2)	0.0239 (19)	−0.0004 (17)	0.0187 (17)	−0.0009 (16)
C24	0.034 (2)	0.024 (2)	0.0229 (19)	−0.0008 (18)	0.0150 (18)	−0.0003 (16)
C25	0.036 (2)	0.028 (2)	0.027 (2)	0.0057 (19)	0.0163 (18)	0.0030 (17)
C26	0.045 (3)	0.023 (2)	0.035 (2)	0.000 (2)	0.028 (2)	0.0049 (18)
C27	0.037 (2)	0.023 (2)	0.034 (2)	−0.0046 (19)	0.025 (2)	−0.0035 (17)
C28	0.031 (2)	0.029 (2)	0.0173 (18)	0.0051 (18)	0.0074 (17)	0.0031 (16)
C29	0.104 (4)	0.024 (2)	0.048 (3)	0.011 (3)	0.051 (3)	0.005 (2)
C30	0.060 (4)	0.064 (4)	0.054 (3)	−0.027 (3)	0.034 (3)	−0.012 (3)
C31	0.089 (6)	0.071 (6)	0.087 (6)	−0.005 (4)	0.027 (4)	−0.019 (4)
C32	0.036 (7)	0.077 (8)	0.069 (7)	−0.008 (6)	0.035 (6)	0.023 (6)
C31'	0.042 (9)	0.045 (9)	0.070 (12)	−0.022 (7)	0.013 (8)	−0.018 (8)
C32'	0.057 (7)	0.047 (6)	0.059 (6)	0.004 (5)	0.026 (5)	0.006 (4)

### Geometric parameters (Å, °)

**Table:**

Zn1—O3	1.940 (2)	C10—C11	1.404 (6)
Zn1—N2	2.019 (3)	C10—C14	1.457 (6)
Zn1—O1	2.031 (2)	C11—C12	1.380 (6)
Zn1—O2	2.034 (3)	C11—H11	0.9500
Zn1—N1	2.054 (3)	C12—C13	1.377 (6)
Zn1—Zn2	3.0134 (6)	C12—H12	0.9500
Zn2—O3^i^	1.940 (2)	C13—H13	0.9500
Zn2—N3	2.018 (3)	C14—H14	0.9500
Zn2—O1	2.025 (2)	C15—C20	1.390 (5)
Zn2—O2	2.036 (3)	C15—C16	1.408 (6)
Zn2—N4	2.064 (3)	C16—C17	1.398 (6)
Cl3—O5	1.403 (4)	C17—C18	1.384 (6)
Cl3—O8	1.405 (4)	C17—H17	0.9500
Cl3—O7	1.429 (3)	C18—C19	1.373 (7)
Cl3—O6	1.462 (4)	C18—H18	0.9500
O1—C9	1.316 (4)	C19—C20	1.384 (6)
O2—C23	1.310 (4)	C19—H19	0.9500
O3—C29	1.396 (5)	C20—H20	0.9500
O3—Zn2^i^	1.940 (2)	C21—C22	1.455 (5)
O4—C30	1.204 (6)	C21—H21	0.9500
N1—C28	1.293 (5)	C22—C27	1.401 (5)
N1—C1	1.422 (5)	C22—C23	1.432 (5)
N2—C7	1.287 (5)	C23—C24	1.423 (5)
N2—C2	1.421 (5)	C24—C25	1.413 (5)
N3—C14	1.285 (5)	C24—C28	1.447 (5)
N3—C15	1.435 (5)	C25—C26	1.370 (6)
N4—C21	1.285 (5)	C25—H25	0.9500
N4—C16	1.422 (5)	C26—C27	1.377 (6)
N5—C30	1.335 (6)	C26—H26	0.9500
N5—C32'	1.475 (9)	C27—H27	0.9500
N5—C32	1.477 (11)	C28—H28	0.9500
N5—C31	1.501 (12)	C29—H29A	0.9800
N5—C31'	1.510 (9)	C29—H29B	0.9800
C1—C6	1.402 (5)	C29—H29C	0.9800
C1—C2	1.413 (6)	C30—H30	0.9500
C2—C3	1.399 (6)	C31—H31A	0.9800
C3—C4	1.375 (6)	C31—H31B	0.9800
C3—H3	0.9500	C31—H31C	0.9800
C4—C5	1.381 (7)	C32—H32A	0.9800
C4—H4	0.9500	C32—H32B	0.9800
C5—C6	1.387 (6)	C32—H32C	0.9800
C5—H5	0.9500	C31'—H31D	0.9800
C6—H6	0.9500	C31'—H31E	0.9800
C7—C8	1.459 (5)	C31'—H31F	0.9800
C7—H7	0.9500	C32'—H32D	0.9800
C8—C13	1.415 (6)	C32'—H32E	0.9800
C8—C9	1.417 (5)	C32'—H32F	0.9800
C9—C10	1.431 (5)		
			
O3—Zn1—N2	110.85 (12)	C8—C9—C10	118.6 (3)
O3—Zn1—O1	107.89 (10)	C11—C10—C9	119.2 (4)
N2—Zn1—O1	89.05 (12)	C11—C10—C14	115.1 (4)
O3—Zn1—O2	106.49 (11)	C9—C10—C14	125.6 (4)
N2—Zn1—O2	142.66 (12)	C12—C11—C10	121.8 (4)
O1—Zn1—O2	79.43 (10)	C12—C11—H11	119.1
O3—Zn1—N1	108.52 (11)	C10—C11—H11	119.1
N2—Zn1—N1	81.23 (13)	C13—C12—C11	119.4 (4)
O1—Zn1—N1	143.43 (11)	C13—C12—H12	120.3
O2—Zn1—N1	87.22 (11)	C11—C12—H12	120.3
O3—Zn1—Zn2	97.31 (8)	C12—C13—C8	121.6 (4)
N2—Zn1—Zn2	129.80 (9)	C12—C13—H13	119.2
O1—Zn1—Zn2	41.93 (7)	C8—C13—H13	119.2
O2—Zn1—Zn2	42.26 (7)	N3—C14—C10	125.2 (4)
N1—Zn1—Zn2	128.55 (9)	N3—C14—H14	117.4
O3^i^—Zn2—N3	112.30 (11)	C10—C14—H14	117.4
O3^i^—Zn2—O1	106.91 (10)	C20—C15—C16	120.2 (4)
N3—Zn2—O1	89.22 (12)	C20—C15—N3	124.6 (4)
O3^i^—Zn2—O2	106.50 (11)	C16—C15—N3	115.2 (3)
N3—Zn2—O2	141.19 (11)	C17—C16—C15	119.3 (4)
O1—Zn2—O2	79.53 (10)	C17—C16—N4	124.6 (4)
O3^i^—Zn2—N4	109.14 (11)	C15—C16—N4	116.1 (3)
N3—Zn2—N4	80.73 (13)	C18—C17—C16	119.6 (4)
O1—Zn2—N4	143.75 (11)	C18—C17—H17	120.2
O2—Zn2—N4	86.77 (11)	C16—C17—H17	120.2
O3^i^—Zn2—Zn1	96.64 (8)	C19—C18—C17	120.6 (4)
N3—Zn2—Zn1	129.79 (9)	C19—C18—H18	119.7
O1—Zn2—Zn1	42.11 (7)	C17—C18—H18	119.7
O2—Zn2—Zn1	42.20 (7)	C18—C19—C20	121.0 (4)
N4—Zn2—Zn1	128.13 (9)	C18—C19—H19	119.5
O5—Cl3—O8	110.8 (3)	C20—C19—H19	119.5
O5—Cl3—O7	110.0 (2)	C19—C20—C15	119.2 (4)
O8—Cl3—O7	108.8 (2)	C19—C20—H20	120.4
O5—Cl3—O6	109.4 (2)	C15—C20—H20	120.4
O8—Cl3—O6	107.4 (2)	N4—C21—C22	125.9 (4)
O7—Cl3—O6	110.5 (2)	N4—C21—H21	117.1
C9—O1—Zn2	130.6 (2)	C22—C21—H21	117.1
C9—O1—Zn1	130.6 (2)	C27—C22—C23	119.2 (4)
Zn2—O1—Zn1	95.96 (11)	C27—C22—C21	116.6 (3)
C23—O2—Zn1	132.1 (2)	C23—C22—C21	124.0 (3)
C23—O2—Zn2	132.3 (2)	O2—C23—C24	120.8 (3)
Zn1—O2—Zn2	95.54 (10)	O2—C23—C22	121.0 (3)
C29—O3—Zn2^i^	117.7 (2)	C24—C23—C22	118.2 (3)
C29—O3—Zn1	116.9 (2)	C25—C24—C23	118.8 (4)
Zn2^i^—O3—Zn1	125.25 (13)	C25—C24—C28	116.2 (4)
C28—N1—C1	123.4 (3)	C23—C24—C28	124.9 (3)
C28—N1—Zn1	127.4 (3)	C26—C25—C24	121.9 (4)
C1—N1—Zn1	109.2 (2)	C26—C25—H25	119.1
C7—N2—C2	122.4 (3)	C24—C25—H25	119.1
C7—N2—Zn1	127.3 (3)	C25—C26—C27	119.4 (4)
C2—N2—Zn1	110.1 (2)	C25—C26—H26	120.3
C14—N3—C15	122.1 (3)	C27—C26—H26	120.3
C14—N3—Zn2	128.0 (3)	C26—C27—C22	121.6 (4)
C15—N3—Zn2	109.4 (2)	C26—C27—H27	119.2
C21—N4—C16	123.9 (3)	C22—C27—H27	119.2
C21—N4—Zn2	127.4 (3)	N1—C28—C24	125.6 (3)
C16—N4—Zn2	108.6 (2)	N1—C28—H28	117.2
C30—N5—C32'	126.5 (9)	C24—C28—H28	117.2
C30—N5—C32	114.8 (7)	O3—C29—H29A	109.5
C30—N5—C31	115.8 (7)	O3—C29—H29B	109.5
C32'—N5—C31	112.8 (11)	H29A—C29—H29B	109.5
C32—N5—C31	129.3 (10)	O3—C29—H29C	109.5
C30—N5—C31'	130.5 (7)	H29A—C29—H29C	109.5
C32'—N5—C31'	102.7 (11)	H29B—C29—H29C	109.5
C32—N5—C31'	108.6 (11)	O4—C30—N5	125.5 (5)
C6—C1—C2	119.8 (4)	O4—C30—H30	117.3
C6—C1—N1	124.4 (4)	N5—C30—H30	117.3
C2—C1—N1	115.9 (3)	N5—C31—H31A	109.5
C3—C2—C1	119.3 (4)	N5—C31—H31B	109.5
C3—C2—N2	124.9 (4)	H31A—C31—H31B	109.5
C1—C2—N2	115.8 (3)	N5—C31—H31C	109.5
C4—C3—C2	120.0 (4)	H31A—C31—H31C	109.5
C4—C3—H3	120.0	H31B—C31—H31C	109.5
C2—C3—H3	120.0	N5—C32—H32A	109.5
C3—C4—C5	121.0 (4)	N5—C32—H32B	109.5
C3—C4—H4	119.5	H32A—C32—H32B	109.5
C5—C4—H4	119.5	N5—C32—H32C	109.5
C4—C5—C6	120.5 (4)	H32A—C32—H32C	109.5
C4—C5—H5	119.8	H32B—C32—H32C	109.5
C6—C5—H5	119.8	N5—C31'—H31D	109.5
C5—C6—C1	119.5 (4)	N5—C31'—H31E	109.5
C5—C6—H6	120.3	H31D—C31'—H31E	109.5
C1—C6—H6	120.3	N5—C31'—H31F	109.5
N2—C7—C8	125.6 (4)	H31D—C31'—H31F	109.5
N2—C7—H7	117.2	H31E—C31'—H31F	109.5
C8—C7—H7	117.2	N5—C32'—H32D	109.5
C13—C8—C9	119.2 (4)	N5—C32'—H32E	109.5
C13—C8—C7	114.6 (4)	H32D—C32'—H32E	109.5
C9—C8—C7	125.5 (3)	N5—C32'—H32F	109.5
O1—C9—C8	120.5 (3)	H32D—C32'—H32F	109.5
O1—C9—C10	121.0 (3)	H32E—C32'—H32F	109.5

Symmetry codes: (i) −*x*+1, *y*, −*z*+1/2.
